# Application of intermittent negative pressure on the lower extremity and its effect on macro‐ and microcirculation in the foot of healthy volunteers

**DOI:** 10.14814/phy2.12911

**Published:** 2016-09-14

**Authors:** Øyvind H. Sundby, Lars Øivind Høiseth, Iacob Mathiesen, Jørgen J. Jørgensen, Harald Weedon‐Fekjær, Jonny Hisdal

**Affiliations:** ^1^ Section of Vascular Investigations Division of Cardiovascular and Pulmonary Diseases Department of Vascular Surgery Oslo University Hospital Oslo Norway; ^2^ Faculty of Medicine Institute of Clinical Medicine University of Oslo Oslo Norway; ^3^ Otivio AS Gaustadalléen 21 Oslo 0349 Norway; ^4^ Department of Anesthesiology Oslo University Hospital Oslo Norway; ^5^ Department of Vascular Surgery Oslo University Hospital Oslo Norway; ^6^ Oslo Center for Biostatistics and Epidemiology Research Support Services Oslo University Hospital Oslo Norway

**Keywords:** Arterial blood flow velocity, dorsal pedis artery, intermittent negative pressure, laser doppler fluxmetry, skin blood flow

## Abstract

Intermittent negative pressure (INP) applied to the lower leg and foot may increase peripheral circulation. However, it is not clear how different patterns of INP affect macro‐ and microcirculation in the foot. The aim of this study was therefore to determine the effect of different patterns of negative pressure on foot perfusion in healthy volunteers. We hypothesized that short periods with INP would elicit an increase in foot perfusion compared to no negative pressure. In 23 healthy volunteers, we continuously recorded blood flow velocity in a distal foot artery, skin blood flow, heart rate, and blood pressure during application of different patterns of negative pressure (−40 mmHg) to the lower leg. Each participant had their right leg inside an airtight chamber connected to an INP generator. After a baseline period at atmospheric pressure, we applied four different 120 sec sequences with either constant negative pressure or different INP patterns, in a randomized order. The results showed corresponding fluctuations in blood flow velocity and skin blood flow throughout the INP sequences. Blood flow velocity reached a maximum at 4 sec after the onset of negative pressure (average 44% increase above baseline, *P *< 0.001). Skin blood flow and skin temperature increased during all INP sequences (*P *< 0.001). During constant negative pressure, average blood flow velocity, skin blood flow, and skin temperature decreased (*P *< 0.001). In conclusion, we observed increased foot perfusion in healthy volunteers after the application of INP on the lower limb.

## Introduction

The idea that air pressure can be manipulated to increase peripheral circulation was first described in the mid‐19th century (Murray 1832, 1835; Junod 1833; Clanny 1835). Expanding upon this discovery, Sinkowitz and Gottlieb (1917) published the first account of the application of a “suction‐device” to treat patients with peripheral arterial disease (PAD) in 1917. The device used constant negative pressure to improve peripheral circulation of the lower limbs. Similarly, several investigations published in the 1930s on the use of intermittent negative and positive pressure on patients with PAD obtained promising results. The studies demonstrated increased skin temperature and wound healing in the foot, as well as limb salvage and improved pain management (Herrmann and Reid 1934; Shipley and Yeager 1934; Takáts 1934; Landis and Hitzrot 1935; Conway 1936).

A previous investigation has demonstrated a large increase in vascular resistance and a subsequent fall in blood flow during application of constant negative pressure (Skagen and Henriksen 1983). Increased venous pressure elicits a vasoconstrictor reflex and substantially reduces blood flow in cutaneous, subcutaneous, and skeletal tissues (Henriksen 1976; Henriksen and Sejrsen 1976, 1977; Skagen and Bonde‐Petersen 1982; Skagen and Henriksen 1983).

In the late 1960s, Caro et al. (1968) demonstrated that a rapid application of negative pressure on the forearm resulted in an abrupt and immediate increase in blood flow in the brachial artery. Smyth (1969) found a large and repetitive increase in arterial inflow in the calf and the femoral arteries when applying short oscillations of intermittent negative pressure (INP) to the lower extremities in individuals with peripheral vascular disease. These results suggest that peripheral blood flow can be increased by means of INP with short oscillations of negative pressure applied directly to a limited part of the lower extremity. Recently, Rein et al. (2007) suggested that applying INP using 10 sec of −40 mmHg and 7 sec of atmospheric pressure increased blood flow to the arm. Rein et al. (2007) suggested that by applying negative pressure intermittently, the vasoconstrictor effect of the venoarterial reflex may be circumvented (Rein et al. 2007). Thereby, short negative pressure oscillations may facilitate an acute and repeated increase in arterial and cutaneous blood flow. However, the effects of INP on macro‐ and microcirculation in the foot remain poorly understood.

The aim of the present experimental study was therefore to determine the effects of different patterns of negative pressure on foot perfusion in healthy volunteers. The study was designed to examine the acute hemodynamic changes during four different sequences of negative pressure applied to a lower extremity. On the basis of previous reports, we hypothesized that short periods with INP would elicit an increase in arterial and cutaneous blood flow in the foot compared to no negative pressure.

## Materials and Methods

### Participants

We recruited 25 healthy volunteers after obtaining their written informed consent: 15 men and 10 women. Inclusion criteria were: (1) good general health and fitness with no abnormal cardiovascular findings on clinical examination, (2) no history of drug abuse (including alcohol), and (3) no medication during the course of the study or past 30 days preceding the study. The participants were normotensive (blood pressure < 140/90 mmHg), nontobacco users, and were free of any known cardiovascular, metabolic, or neurological diseases.

The participants were asked to refrain from eating 2 h before the start of the study and from consuming alcohol or caffeine 24 h before the start of study. They were also asked not to take any vitamin supplements 72 h before study or to perform intense physical activity 12 hours before their visit. All participants had a normal ankle‐brachial index (ABI) (≥1.0), pulse volume recordings (PVR) waveforms with amplitude >10 mm, and a brisk upstroke and downstroke with dicrotic notch, indicating good peripheral circulation. The participants were informed about the general nature of the experiment and the length of each sequence, but not the order of interventions. The experimental protocol was approved by the Regional Committees for Medical and Health Research Ethics in Norway (protocol number: 2014/1967) and performed in accordance with the Declaration of Helsinki.

### Experimental design

**Figure 1:**
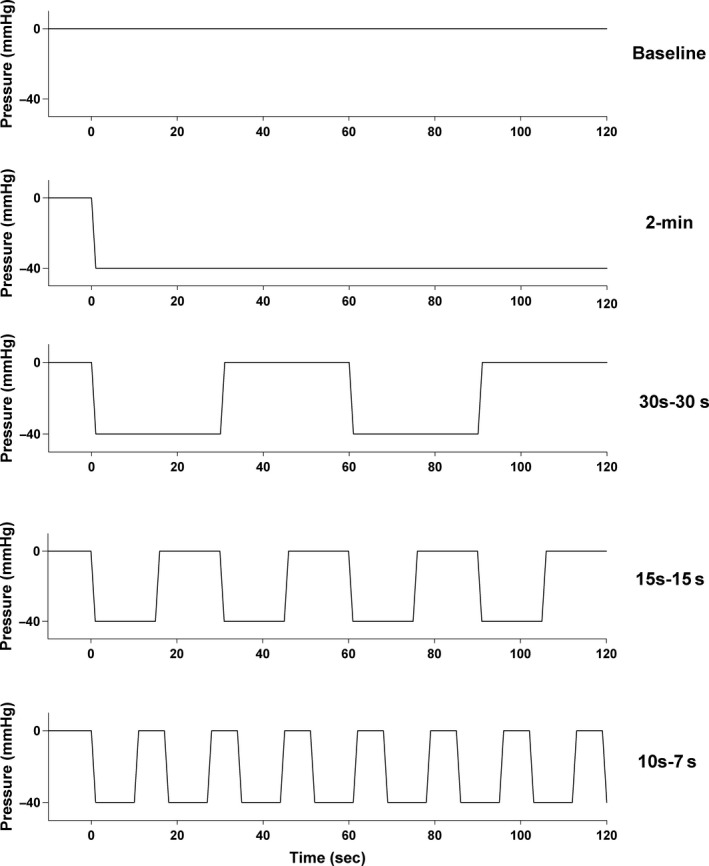
Illustration of the five 2‐min sequences. From top: Baseline, 2‐min constant negative pressure, 30 sec‐30 sec, 15 sec‐15 sec, and 10 sec‐7 sec. All negative pressure oscillations: −40 mmHg.

The participants were comfortably clothed and seated in an armchair for 30 min before the experiment started. After 2‐min baseline registrations with no pressure manipulation (atmospheric pressure, 760 mmHg), four sequential INP cycles with −40 mmHg (compared to atmospheric pressure) were applied for 2‐min in a randomized order: (1) 2‐min constant negative pressure, (2) 30 sec negative pressure/30 sec atmospheric pressure, (3) 15 sec negative pressure/15 sec atmospheric pressure, or (4) 10 sec negative pressure/7 sec atmospheric pressure (Fig. 1). Randomization was performed by generating random numbers in Microsoft Excel (Microsoft Office 2010 for Windows; Microsoft, Redmond, WA). After a wash‐out period of minimum 5 min, the same protocol was repeated, giving participants two sets of measurements of all five 2‐min sequences. Randomization limited the order effect and a 5 min wash‐out period between sets reduced any potential carry‐over effect. All investigations were conducted in a quiet, temperature‐controlled environment (24.6 ± 1.5°C) to reduce sympathetic stress that could create artifacts (Thoresen and Walloe 1980).

### Anthropometric measurements

After taking measurements of stature (Modell MZ 10023, ADE, Hamburg, Germany) and body weight (Seca medical scales, Seca 877, Hamburg, Germany), we obtained ABI measurements with the participant in a supine position, using a continuous‐wave hand‐held 8 MHz Doppler blood velocity detector (Macrolab, STR Teknikk, Aalesund, Norway). We used a blood pressure cuff to measure systolic blood pressure to the nearest 2 mmHg in the brachial artery of both arms. For the legs, we used a Doppler detector to measure systolic blood pressure to the nearest 2 mmHg in the dorsalis pedis artery. We calculated the ABI numerator by dividing the higher ankle systolic pressure (obtained from the dorsalis pedis arteries) by the higher of the two arm systolic pressures (Aboyans et al. 2012). Last, we took pulse‐volume recordings (MacroLab, STR Teknikk, Aalesund, Norway) in the lower limbs by means of an air‐plethysmography cuff placed on each participant's distal ankle (Bo et al. 2013).

### Signal acquisition and analysis

We measured arterial blood flow velocity (cm∙sec^−1^) in the dorsalis pedis/posterior tibial artery with a 10 MHz pulsed Doppler probe (SD‐50; GE Vingmed Ultrasound, Horten, Norway). The ultrasound beam was positioned centrally at either the dorsum pedis or posterior to the medial malleolus of the ankle, depending on where we obtained the highest velocity signal. We did not measure the diameter in the dorsal pedis/tibialis posterior artery, but previous research has shown that the pulsatile diameter in small arteries is very small (Eriksen 1992). We therefore assumed that the changes in observed arterial blood flow velocity during negative pressure oscillations reflect changes in blood flow in the foot artery and not only changes in the artery diameter.

We used laser Doppler fluxmetry (LDF; Periflux PF 4000; Perimed AB, Järfälla, Sweden) to measure acral skin blood flow, giving a semi‐quantitative real‐time measurement of cutaneous peripheral microcirculation expressed in arbitrary units (AU) (Sarnik et al. 2007). After preparing the skin with an alcohol swab, we attached LDF probes (404‐1; Perimed AB, Järfälla, Sweden) bilaterally to the skin of the pulps of the big toes.

We recorded finger arterial pressure continuously from the third finger of the right hand using a photoplethysmographic volume‐clamp method (Finometer; FMS Finapres Medical Systems BV, Amsterdam, The Netherlands). At the beginning of each study, Finapres pressures were calibrated with measurements from an automated sphygmomanometer connected to a patient monitor (Solar 8000i; GE‐Marquette Medical Systems, Inc., Milwaukee, WI). Skin temperature was continuously measured within the boot on the foot, in close proximity to the dorsalis pedis artery, using an Analog Devices AD590 temperature transducer (STR Teknikk, strteknikk.no, Aalesund, Norway).

Analog signals for all measurements were sampled at 300 Hz and averaged for each heartbeat gated by the R‐waves of the 3‐lead ECG using custom‐made software (REGIST 3; Morten Eriksen, University of Oslo, Oslo, Norway). In all experiments, measurements were carried out by the same researcher. The participants were instructed to avoid moving and talking throughout the sampling period.

### Application of intermittent negative pressure

**Figure 2:**
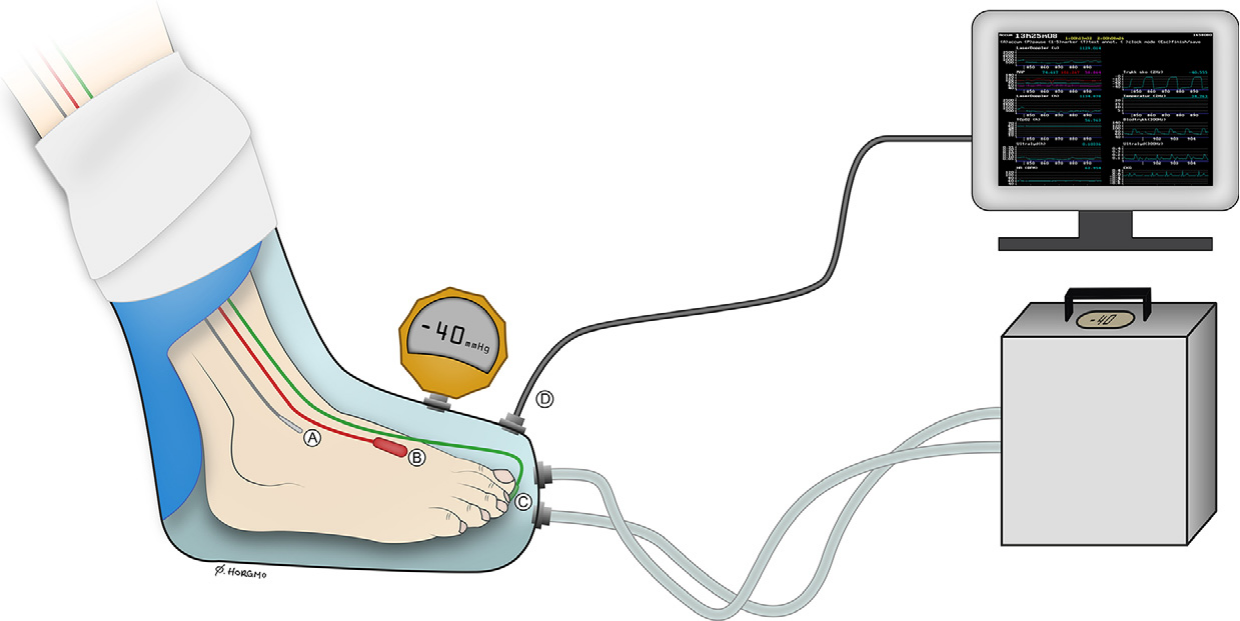
Illustration of the test setup with probes attached to the foot. The participant's leg was placed in a custom‐made chamber interfaced with the pressure control system. The chamber acted as a vacuum chamber, by sealing around the participant's leg below the knee. The left leg was placed outside the vacuum chamber, acting as a control in atmospheric pressure. (A) Skin temperature probe; (B) Ultrasound Doppler probe; (C) Laser Doppler flux probe; (D) Pressure transducer from boot interfaced with the computer. An additional external pressure device was connected to the boot in order to calibrate boot pressure. Illustration: Øystein H. Horgmo, University of Oslo.

During the test runs, the participants were asked to sit comfortably in an armchair with an approximate angle of 130° in their knee and hip joints **(**Fig. 2). The participants' body and legs were covered with a blanket. After attachment of probes, both of the participants' feet were covered with loose, non‐elastic wool socks to keep their feet warm and under thermoneutral conditions during the course of the study. The right leg was placed in a rigid molded polyethylene boot coupled to a pressure control system (FlowOx^™^; Otivio AS, Oslo, Norway), with the contralateral leg acting as a control. The boot had internal padding to allow insertion of a leg with probes and prevent pressure points on the leg and skin. The boot was sealed just below the knee with a thermoplastic elastomer (TPS‐SEBS) to allow for application of negative pressure. Pressure was continuously monitored throughout the study using a calibrated pressure transducer (Fluke, 700G Series, Everett, WA) attached to the boot and analyzed in REGIST 3 (Fig. 2).

### Tolerability of negative pressure

At the end of each experiment, the user was asked to rate their level of comfort during INP using a verbal numerical pain rating scale 0–10, ranging from no pain or discomfort (0) to worst imaginable pain (10).

### Statistical analysis

All data are presented as mean (95% CI) unless otherwise stated. Hemodynamic measures were captured in a baseline sequence without manipulation of pressure prior to each randomization. We repeated all sequences, except the baseline, in a randomized order. Effects of the four different sequences over time compared to baseline were evaluated in a regression model, by assigning variables to each of the different sequences. Due to data correlation within participants, we analyzed the data in a linear mixed regression model with participants as a random effect to account for individual differences, using the R nlme package (R Foundation for Statistical Computing, Vienna, Austria). The intercept of the regression model gave the estimate of flow in the baseline sequence. The effects of each variable gave the differences from the baseline sequence. Confidence intervals for the different sequences analyzed in the mixed regression model were extracted using the “glht” function of the “multcomp” package in R (R Foundation for Statistical Computing, Vienna, Austria).

To estimate the maximal effect of INP on blood flow, we aggregated the first 10 sec of all the negative pressure oscillations in both sequences. To avoid using the same data to both locate the maximal blood flow velocity and estimate its magnitude, we used the first set to locate the time of maximal blood flow velocity and the second set to estimate its magnitude, and vice versa. For the 10 sec‐7 sec, 15 sec‐15 sec, and 30 sec‐30 sec‐seqences, we evaluated maximum blood flow velocity using only the first negative pressure period within each sequence. To evaluate the effects of pressure over time within each sequence, we rounded the time of each observation (heartbeat) to the nearest second. Variables were assigned to each second, and effects of pressure over time within sequences were evaluated and compared to the values from the first second of each sequence. For all statistical tests, a two‐tailed probability level of *P *< 0.05 was considered statistically significant.

## Results

Twenty‐five healthy volunteers (15 males, 10 females) were included in the study. Due to small movements during suction, adequate, high‐quality Doppler signals could not be obtained from two participants. Consequently, the results of 23 participants were available for analysis (15 males, 8 females). Table 1 presents the 23 participants' anthropometric data. On the verbal numeric rating scale (0–10), one participant rated discomfort at 1 while the rest rated 0 (Table 1).

**Table 1:** Demographic and anthropometric data, *n* = 23^c^

Variables^a^	
Age (year)	27 (7.6)
Body mass (kg)	73.5 (11.8)
Stature (cm)	179 (0.1)
BMI (kg∙m^−2^)	22.8 (2.0)
Ankle‐brachial index (%)	107.0 (0.1)
PVR right (mm)	20.9 (9.4)
PVR left (mm)	18.3 (7.2)
Systolic blood pressure (mmHg)	113.7 (10.0)
Diastolic blood pressure (mmHg)	70.7 (6.8)
Mean arterial pressure (mmHg)	85.0 (7.1)
Verbal numeric pain scale (0–10)^b^	0.0 (0–10)
Aerobic exercise (h∙week^−1^)	5.2 (4.0)
Training frequency (times∙week^−1^)	5.8 (3.6)

a Values are mean (SD).

b Values are median (min‐max).

c 15 males and 10 females.

BMI, body mass index; PVR, pulse volume recording.

### Blood flow velocity in the foot over whole 2‐min registration period

**Figure 3:**
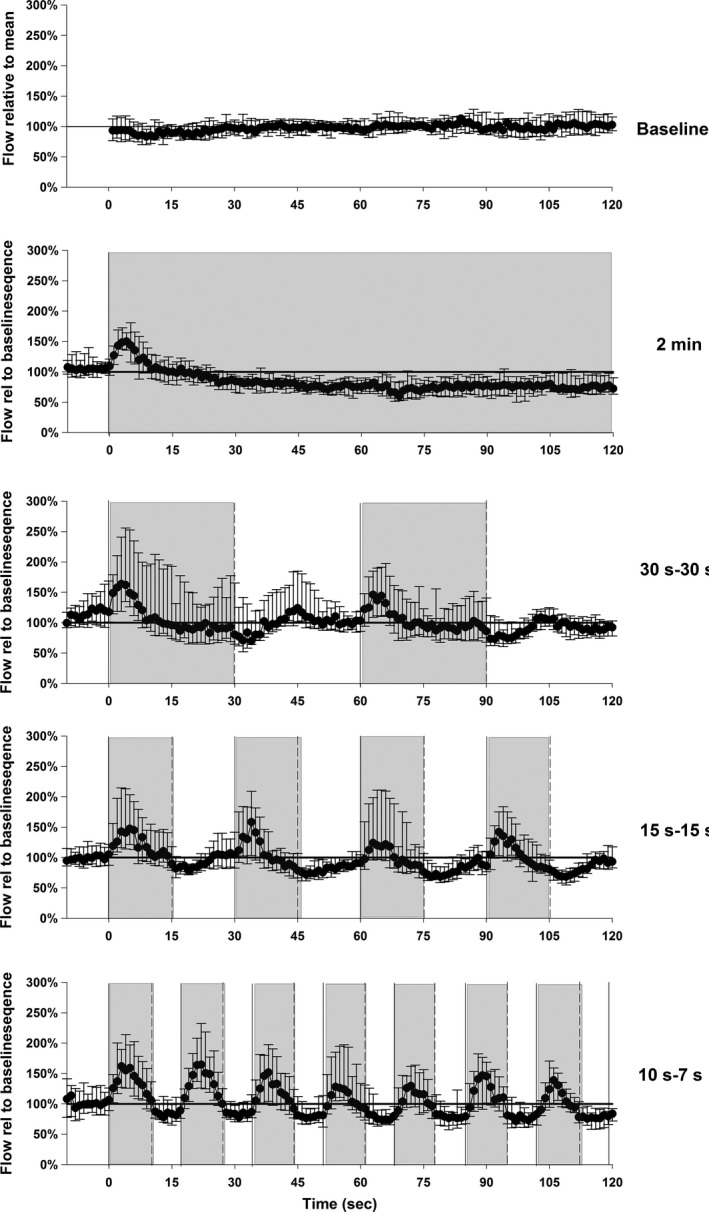
Blood flow velocity in the dorsal pedis artery/posterior tibial artery (ultrasound Doppler), during 2‐min period in atmospheric pressure (baseline) and in four different sequences of negative pressure. The data are normalized for each subject to the average of the baseline period. Values are median and 25th and 75th percentiles. Grey color indicates negative pressure.

Median baseline arterial blood flow velocity captured during a 2‐min registration period was 8.9 (6.4–11.4) cm∙sec^−1^. Compared to the 2‐min baseline sequence (atmospheric pressure), blood flow velocity was increased in the 10 sec‐7 sec and 30 sec‐30 sec sequences to 9.6 (7.1–12.1) and 10.1 (7.6–12.6 cm∙sec^−1^), respectively (both *P *< 0.001). Blood flow velocity did not significantly change during the 15 sec‐15 sec sequence: 8.8 (6.3–11.2) cm∙sec^−1^ (*P *= 0.24). A reduction in blood flow velocity compared to the 2‐min baseline was observed in the sequence with 2‐min constant negative pressure: 7.8 (5.3–10.2) cm∙sec^−1^ (*P *< 0.001). Beat‐by‐beat plots of the median blood flow velocities are presented in Figure 3.

**Figure 4:**
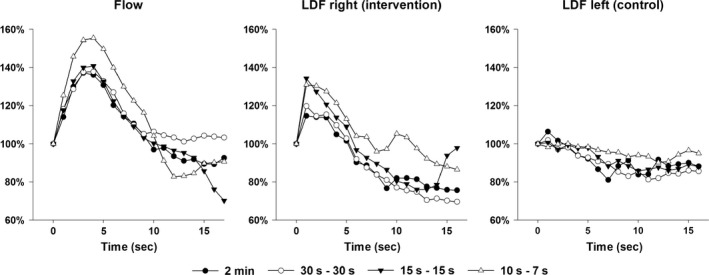
Effects of time after onset of negative pressure comparing different sequences. All INP‐sequences were aggregated, and estimated by assigning variables to each second in a linear mixed regression model. All values are relative to the baseline sequence (100%). ‘‘LDF right’’ is measured on the foot with negative pressure, whereas “LDF left” is measured on the contralateral foot (control leg). Flow, Blood flow velocity; LDF, laser Doppler fluxmetry.

**Figure 5:**
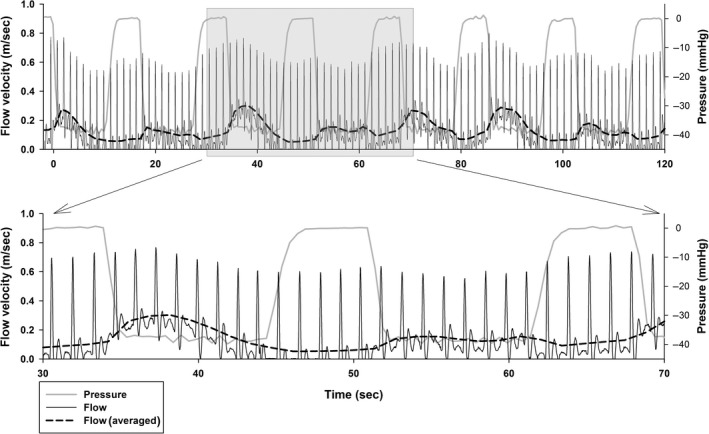
A representative 10 sec‐7 sec sequence from one participant. Blood flow velocity per heartbeat (ultrasound Doppler) is displayed in solid black lines. The dashed lines show the trend line for average blood flow velocity per heartbeat. Both are measured along the left *y*‐axis. Boot pressure grey lines, right *y*‐axis. Upper panel is an entire 120 sec sequence, shaded area is zoomed in lower panel.

Peak blood flow velocity occurred after about 4 sec with an increase of 44 (55–33) % from the onset of negative pressure (*P *< 0.001) (Fig. 4). The blood flow velocity response to INP varied between and within each participant (range: ~40–200% increase in blood flow velocity). See Figure 5 for a representative blood flow velocity response to INP in one participant.

### Laser Doppler fluxmetry and skin temperature

#### Effects of different sequences averaged over the entire 2‐min registration period

The mean baseline flux was 74 (49–110) AU. In the right (intervention) foot, all sequences except the 2‐min sequence were associated with increases in flux (*P *< 0.001), with the largest increases during the 10 sec‐7 sec sequence with a mean of 90 (67–134) AU (*P *< 0.001). In the left (control) foot, all sequences were associated with a decrease in flux compared to the baseline sequence (*P *< 0.001). Flux increased simultaneously with blood flow velocity. Maximum flux was reached about 2 sec after the onset of negative pressure, with the highest values attained during the 10 sec‐7 sec and 15 sec‐15 sec sequences (Fig. 4).

Mean skin temperature during the 120 sec sequences was 26.4 (25.3–27.5)°C at baseline, 26.5 (25.4–27.7) °C in the 10 sec‐7 sec, 26.5 (25.4–27.6)°C in the 15 sec‐15 sec, 26.5 (25.4–27.7)°C in the 30 sec‐30 sec, and 25.5 (24.4–26.6)°C in the 2‐min sequences.

### Central hemodynamics

There were only minor and no clinically significant changes in heart rate (HR) and mean arterial pressure (MAP) between the different pressure sequences. Mean HR at baseline was 62 (58–67) beats∙min^−1^. There was a difference of less than two heartbeats between baseline, INP, and constant negative pressure sequences: 10 sec‐7 sec: 61.5 (61.3–61.7) beats∙min^−1^, *P *< 0.001; 15 sec–15 sec: 60.9 (60.7–61.1) beats∙min^−1^ (*P *< 0.001); 30 sec‐30 sec: 62.1 (61.9–62.3) beats∙min^−1^ (*P *= 0.42); 2 min: 62.4 (62.2–62.6) beats∙min^−1^ (*P *< 0.001).

MAP was 67 (57–77) mmHg at baseline. During the negative pressure sequences, MAP increased by less than 2.5 mmHg: 10 sec–7 sec: 68.9 (68.7–69.1 mmHg (*P *< 0.001); 15 sec‐15 sec, 69.6 (69.4–69.9) (*P* < 0.001); 30 sec–30 sec: 69.1 (68.9–69.3) mmHg (*P *< 0.001); 2 min: 69.4 (69.2–69.7) mmHg *(P *< 0.001).

## Discussion

The main finding in this study was that in all sequences, arterial blood flow velocity increased after the onset of negative pressure, with peak flow after 4 sec. On average, we observed a peak increase of 44% compared to baseline. In addition, we observed a corresponding increase in acral skin blood flow. Last, arterial blood flow velocity, skin blood flow, and skin temperature decreased following the application of constant ambient negative pressure. The findings support our hypothesis that the application of oscillating negative pressure, applying brief periods of intermittent negative and atmospheric pressure, increases arterial blood flow velocity and skin blood flow in the foot of healthy participants.

Our results support previous reports where the same INP sequence was applied to the arms in combination with temperature‐controlled water to influence body core temperature (Rein et al. 2007, 2014). In the first study, Rein et al. (2007), demonstrated that combining warm water and oscillations of 10 sec of negative pressure and 7 sec of atmospheric pressure to an upper extremity was more efficient than conventional forced‐air heating in preventing hypothermia during laparotomy. In their second study (Rein et al. 2014), the authors exposed volunteers to passive heat stress while testing the effect of two portable negative pressure devices on core body temperature. The authors demonstrated significantly increased cooling efficacy using the 10 sec‐7 sec INP‐sequence in combination with a temperature of 24°C that covered the whole arm, compared to a device that applied constant negative pressure and cooling temperature of 19°C sealed around the hand (CoreControl, Avacore Inc. Ann Arbor, MI). Unfortunately, Rein et al. (2007, 2014) did not measure arterial lood flow velocity and acral skin in the arm exposed to INP. In this study, we observed an increase in blood flow velocity and acral skin blood flow during each negative pressure oscillation. In addition, we were also able to record a small increase in skin temperature on the dorsum of the foot during all INP sequences. Although the 2‐min INP sequences applied in this study increased skin temperature while the constant negative pressure decreased skin blood flow, these changes were small and probably clinically insignificant. Interestingly, Herrmann and Reid (1934) reported increased skin temperature at rest in PAD patients treated with intermittent negative and positive pressure several hours per day for 2 weeks or more. Skin temperature is an indirect measure of improved skin blood flow, and together with the increased LDF, these observations suggest that INP improved blood flow in the small vessels of the skin.

Smyth (1969) found increased arterial inflow in the leg arteries of patients after 6 weeks’ application of an INP sequence of 15 sec of negative pressure and 15 sec of atmospheric pressure. In the same study, the researchers also observed increased femoral arterial blood flow velocity during application of a 15 sec‐15 sec sequence (Smyth 1969). When comparing the ultrasound measures in the calves of 11 patients with severe peripheral vascular disease before and after 6 weeks of twice‐weekly INP treatment, the authors observed an increased average resting blood flow of 282% compared to pretreatment (Smyth 1969). The Smyth study also reported increased peak reactive hyperemia after occlusion by 300% compared to pretreatment. In our study, the 15 sec‐15 sec sequence generated greater fluctuations in blood flow velocity than the baseline sequence, the 2‐min pressure sequences, and the 30 sec‐30 sec sequence (Fig. 3). The average blood flow velocity in the 15 sec‐15 sec, however, was not significantly different from baseline (Fig. 3). In general, the difference in average blood flow velocity between baseline and during INP sequences (10 sec‐7 sec and 30 sec‐30 sec) in this study was minor, and probably not clinically significant. One possible explanation for this finding may be that the observed increase in flow during the first seconds after onset of negative pressure was counteracted by a reduction, starting 4 sec‐5 sec after the onset of negative pressure (Figs. 3 and 4). It is possible that the clinical effects of 6 weeks of INP therapy on wound healing, blood flow and walking distance described by Smyth (1969) were due to the pulsatile shears stress or other biochemical effects from fluctuations in blood flow and not the average increase in blood flow.

There have been many recent studies describing the importance of arteriolar vasomotion and capillary flow motion for nutrition and oxygen supply to the tissues (Stucker et al. 1998; Li et al. 2006; Thorn et al. 2009, 2011). Although the mechanism by which this occurs is still largely unknown, flowmotion is regarded as more essential than average flow in determining adequate tissue perfusion (Stefanovska 2009). In our study, the frequent oscillations of negative pressure elicited pulsatile flow, indicated by large fluctuations in arterial and skin blood flow during the negative pressure cycles. Such fluctuations have been linked to beneficial effects in tissue perfusion. First, the dynamic rhythmic fluctuations in capillaries and arteries (flowmotion) are believed to be an important factor in tissue oxygenation (Tsai and Intaglietta 1993; Aalkjaer et al. 2011). Second, the high‐flow velocity oscillations are believed to induce sufficient shear stress on the endothelium to stimulate the release of biochemical mediators. For example, increased pulsatile flow—in contrast to cells exposed to steady shear stress—has been demonstrated to increase prostacyclin (Frangos et al. 1985), endothelial‐derived relaxing factor (Cooke et al. 1990), platelet‐derived growth factor (Hsieh et al. 1991), and tissue‐type plasminogen activator (Nollert et al. 1991) in cell culture models. Also, a randomized trial on patients with peripheral arterial disease showed that combining intermittent positive and negative pressure improved walking distance and the adenosine diphosphate threshold for platelet aggregation (Mehlsen et al. 1993). Although care should be exercised in interpreting the results of this study, the enhanced peripheral circulation we observed with the use of INP may have clinical implications. Nevertheless, further studies are needed to elucidate any potential mechanisms and clinical implications of the pulsatile flow observed during INP.

In this study, the 2‐min constant negative pressure sequence induced both reduced arterial blood flow velocity (Fig. 3) and pulp skin blood flow, which was accompanied by a small reduction in skin temperature. This reduction in arterial blood flow in a limb when exposed to constant negative pressure has been associated with the local sympathetic venoarteriolar reflex (Henriksen 1976; Skagen and Henriksen 1983).

This study demonstrated that repeated application of mild INP using −40 mmHg evoked a repetitive increase in local macro‐ and microcirculation with only a small, probably clinically irrelevant, increase in mean arterial pressure in healthy participants. Compared to the baseline, heart rate was only 0.5 beats∙min^−1^ and 1.1 beats∙min^−1^ lower during 10 sec‐7 sec and 15 sec‐15 sec sequences, respectively, while it increased <0.5 beats∙min^−1^ for the 30 sec‐30 sec and 2‐min sequences. These differences were small and probably clinically insignificant. It is possible that the small increase in systemic blood pressure is caused by an increase in total peripheral resistance as a result of repeated activation of the venoarteriolar reflex at the onset of negative pressure. In this study, none of the participants reported any pain or unpleasant sensations during INP application. This was confirmed by a verbal numerical rating score close to zero. Consequently, the application of INP may facilitate repetitive arterial inflow, while at the same time avoiding the contact rash and skin abrasion often associated with compression (Moran et al. 2015).

### Limitations to the study

There are some limitations to be addressed. First, we did not design this study to monitor venous pressure, and are therefore not able to elaborate about the mechanisms behind the changes in blood flow observed during application of negative pressure in the foot. Further investigations are therefore warranted to study the exact mechanisms. Second, in this study we did not measure the diameter of the dorsal pedis/tibialis posterior artery during INP. However, we found a similar response between blood flow velocity and acral skin blood flow, measured with laser Doppler flux. Eriksen (1992) found pulsatile diameter in small arteries to be very stable, and Hisdal et al. (2001) observed a strong correlation between acral skin blood flow, measured by the laser‐Doppler technique, and blood flow velocity. We therefore assume that the changes observed in the blood flow velocity during INP reflect changes in blood flow in the foot artery, and not changes in artery diameter. Last, this study was conducted on healthy volunteers, and care should be exercised when extrapolating the results to different patient groups, such as those with peripheral arterial disease, diabetes or a dysfunctional autonomic nervous system, that is, following spinal cord injury.

## Conclusion

This is the first study to describe the effects of INP on skin blood flow and arterial blood flow velocity. To the best of our knowledge, it is also the first to compare different sequences of negative pressure oscillations on lower limb perfusion. This study found that application of frequent mild intermittent negative pressure (INP) of −40 mmHg in the foot in healthy volunteers induced rhythmical fluctuations in blood flow and increased both arterial blood flow velocity and skin blood flow. The significance of the observed transient increase in peripheral circulation for oxygen supply and tissue proliferation requires further investigation to examine the clinical effects of repetitive use. Future investigations should also examine the working mechanisms of INP.

## Conflict of Interest

The study was supported in part by Otivio AS. ØHS is a PhD student at University of Oslo employed by Otivio. Otivio AS owns and has the commercial rights to the INP technology used in the study. IM is the CEO, a co‐founder and a shareholder of Otivio AS. None of the other authors have any personal conflicts of interest – financial or otherwise. The authors alone are responsible for the content and writing of the paper.
